# Supplementary material for: TCDD dysregulation of lncRNA expression, liver zonation and intercellular communication across the liver lobule
Source: bioRxiv. 2023 Jan 8:2023.01.07.523119. Preprint. [Version 1] doi: 10.1101/2023.01.07.523119 (PMC9881922; doi:10.1101/2023.01.07.523119)

bioRxiv preprint doi: https://doi.org/10.1101/2023.01.07.523119; this version posted January 8, 2023. The copyright holder for this preprint (which was not certified by peer review) is the author/funder, who has granted bioRxiv a license to display the preprint in perpetuity. It is made available under aCC-BY-NC-ND 4.0 International license.

Fig. S1

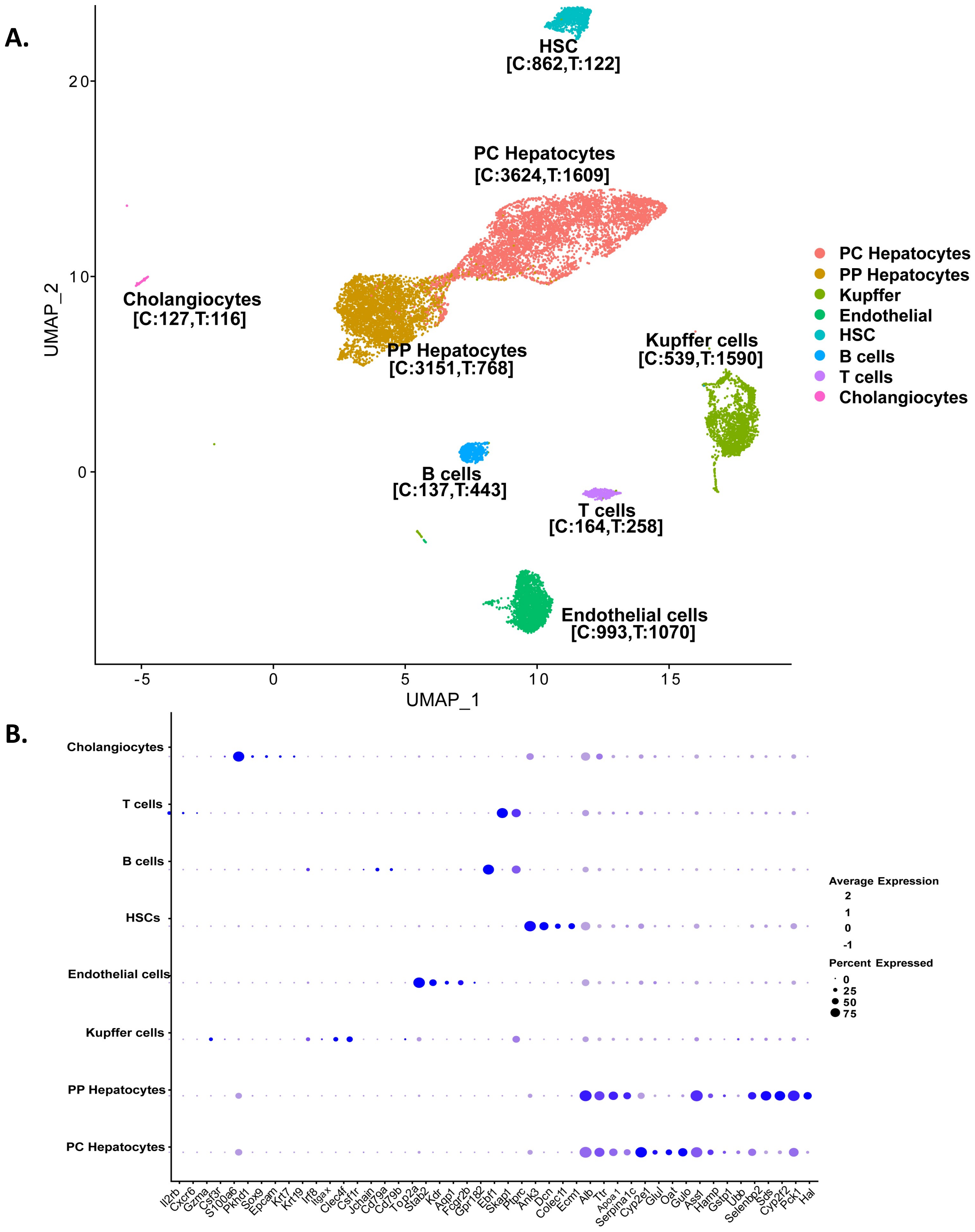

bioRxiv preprint doi: https://doi.org/10.1101/2023.01.07.523119; this version posted January 8, 2023. The copyright holder for this preprint (which was not certified by peer review) is the author/funder, who has granted bioRxiv a license to display the preprint in perpetuity. It is made available under aCC-BY-NC-ND 4.0 International license.

Fig. S2

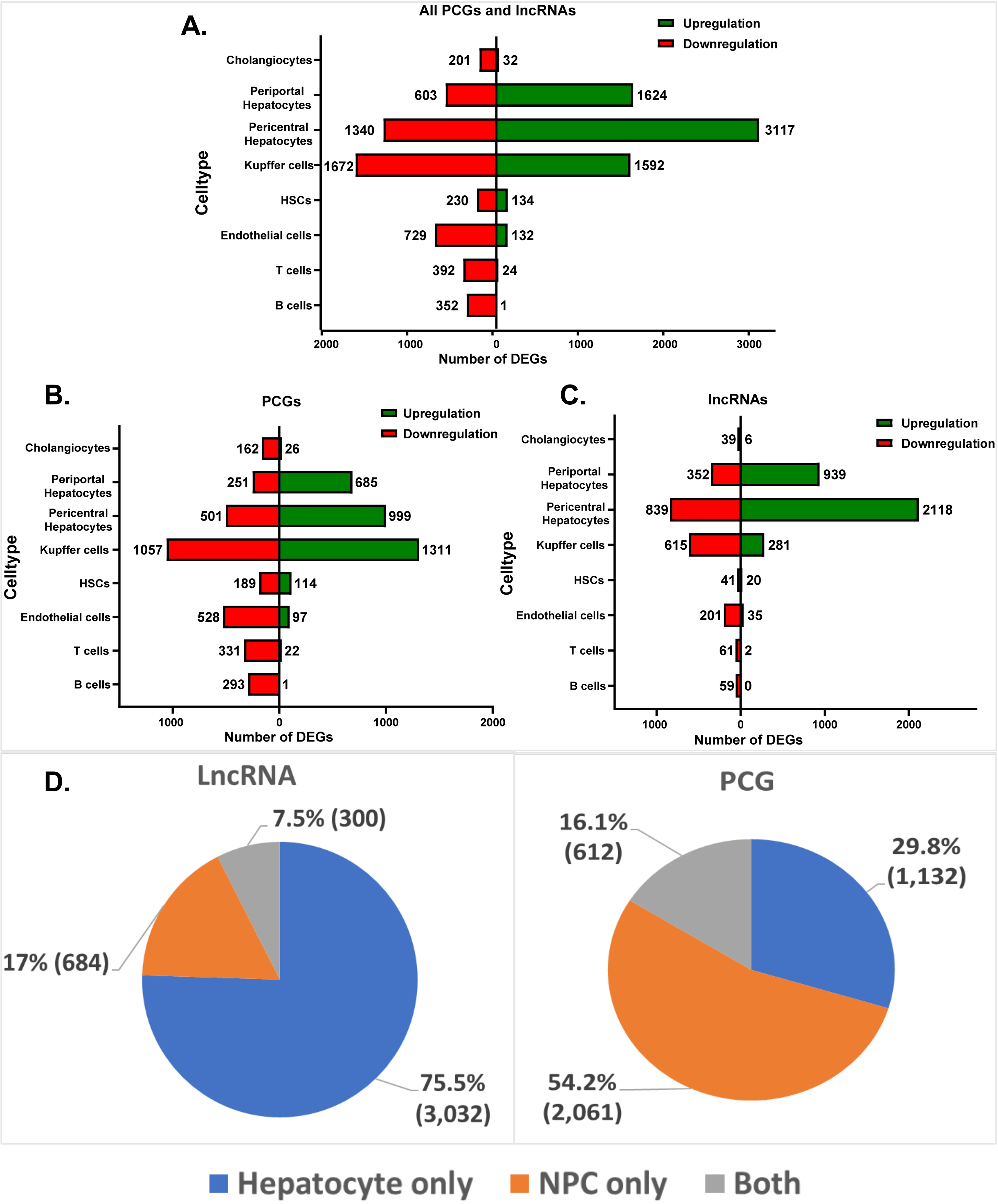

Fig. S3AB

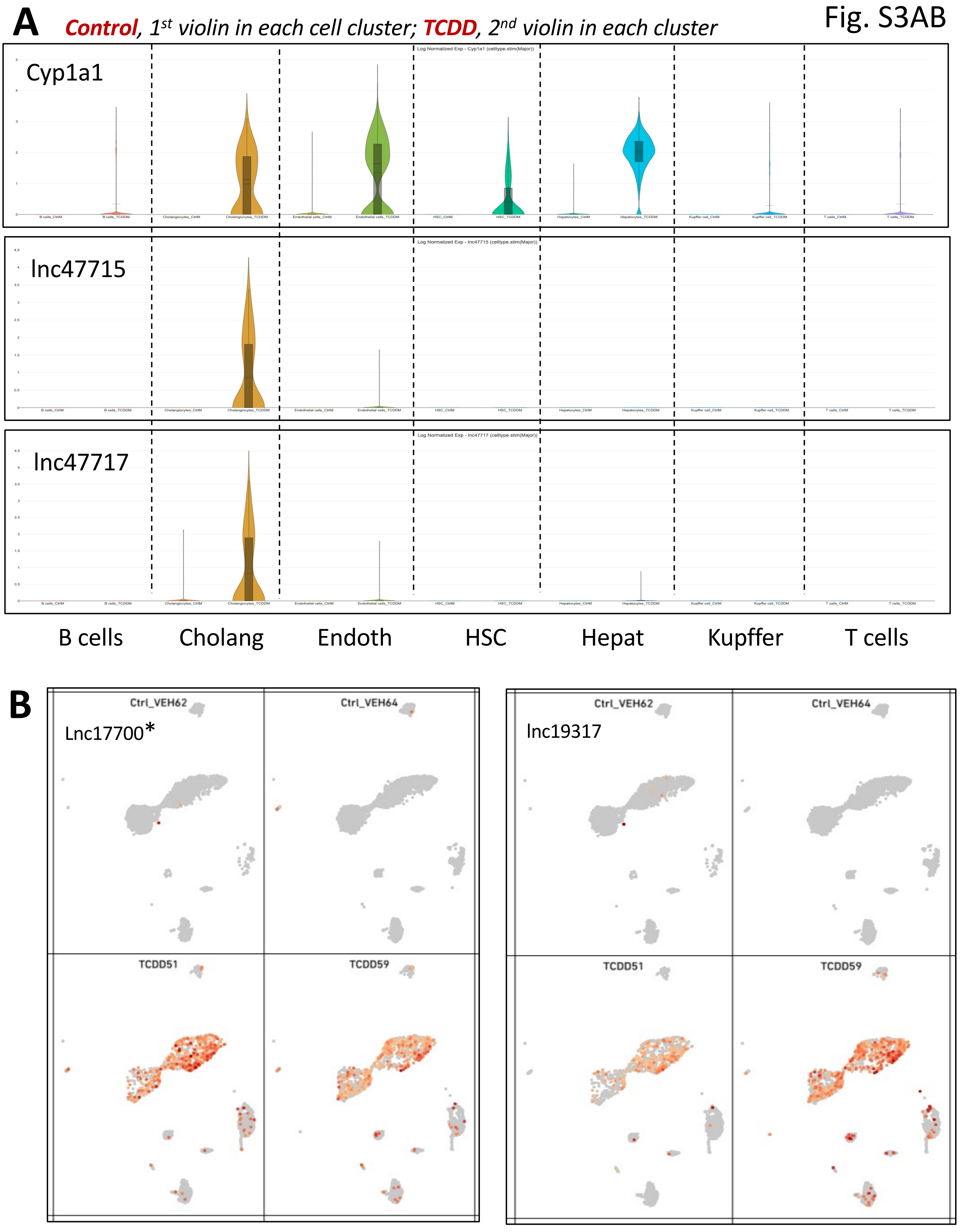

Top two sub-panels: control liver (n=2);
bottom two sub-panels: TCDD liver (n=2)

Fig. S3C

C

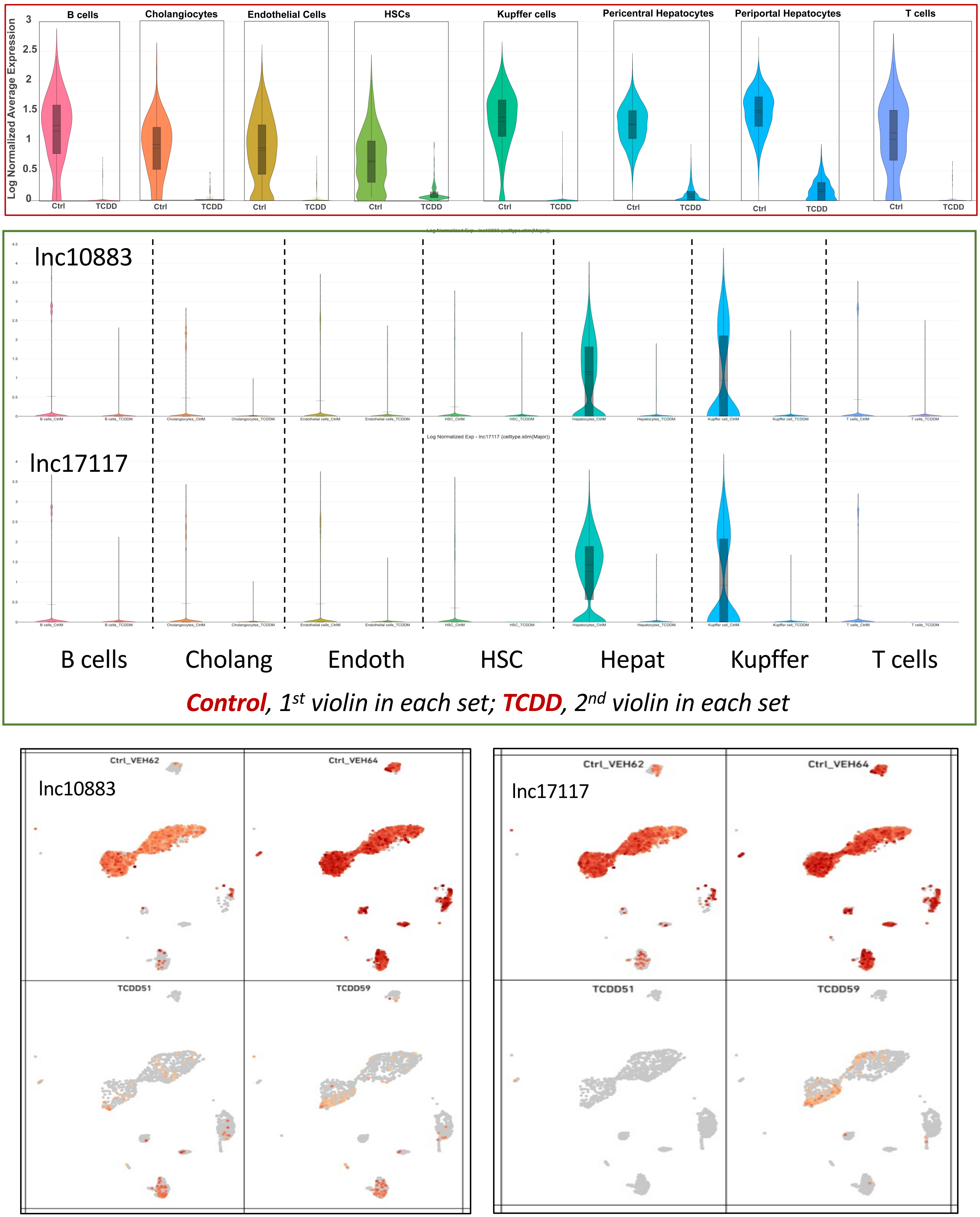

Top two sub-panels: control liver (n=2); bottom two sub-panels: TCDD liver (n=2)

Fig. S4

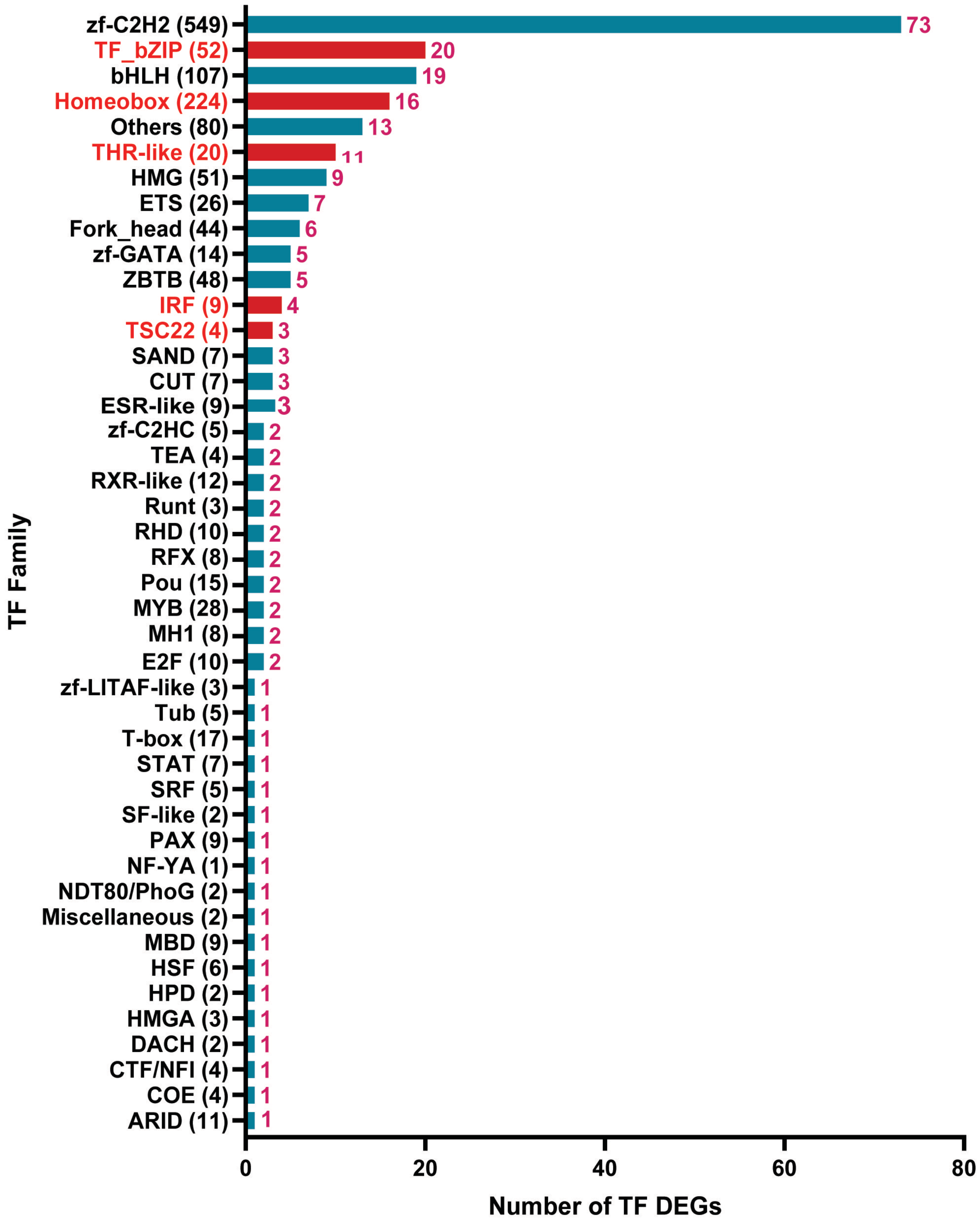
zf-C2H2 (549) 73
TF_bZIP (52) 20
bHLH (107) 19
Homeobox (224) 16
Others (80) 13
THR-like (20) 11
HMG (51) 9
ETS (26) 7
Fork_head (44) 6
zf-GATA (14) 5
ZBTB (48) 5
IRF (9) 4
TSC22 (4) 3
SAND (7) 3
CUT (7) 3
ESR-like (9) 3
zf-C2HC (5) 2
TEA (4) 2
RXR-like (12) 2
Runt (3) 2
RHD (10) 2
RFX (8) 2
Pou (15) 2
MYB (28) 2
MH1 (8) 2
E2F (10) 2
zf-LITAF-like (3) 1
Tub (5) 1
T-box (17) 1
STAT (7) 1
SRF (5) 1
SF-like (2) 1
PAX (9) 1
NF-YA (1) 1
NDT80/PhoG (2) 1
Miscellaneous (2) 1
MBD (9) 1
HSF (6) 1
HPD (2) 1
HMGA (3) 1
DACH (2) 1
CTF/NFI (4) 1
COE (4) 1
ARID (11) 1
TF Family
0
20
40
60
80
Number of TF DEGs

Fig. S5

Abca6,Abcc6,Ablim3,Acad11,Acat1,Acot12,Acox2,Adipor2,Akr1c20,Aldob,Apob,Bckdha,Bdh1,
Cep85,Cyp4f15,Ddc,Deptor,Dgat2,Dhcr24,Dhtkd1,Eng,Ephx2,Etnppl,Fah,Fbp1,Galm,Gclc,Gm15622,
Gpam,Hgd,Hjv,Immp2l,Inca1,Klf12,Klf15,Ldb2,Ldlr,lnc10476,lnc11040,lnc12776,lnc12905,lnc13247,
lnc1345,lnc1360,lnc1416,lnc14332,lnc16799,lnc17060,lnc1884,lnc22198,lnc2936,lnc31482,lnc40890,
lnc5389,lnc5718,lnc6251,lnc7720,Loxl2,Lpcat3,Lpin2,Mia2,Mlxipl,Msra,Mtarc1,Mthfd1,Nlrp6,Oaf,Optn,
Pbld1,Pcyt2,Pde4dip,Pecr,Pipox,Ppp1r3b,Prickle2,Proc,Prodh2,Prox1,Rapgef4,Rbp4,Rtp3,Sec24a,Sema4g,
Serpinf2,Slc10a1,Slc25a13,Slc25a20,Slc25a21,Slc27a2,Slc39a14,Stard10,Xylb,Ankrd33b,Arhgap31,Arid5b,
Arrb1,Bmp5,Bmp6,Cd38,Cpt1a,Creg1,Ctdspl,Dab2,Dcn,Ddi2,Dennd2a,Elk3,Ets1,Etv1,Fbn1,Haao,Hdac7,
Hebp1,Heyl,Hivep3,Hoxb4,Hsd17b4,Ifi203,Igf1,Igf1r,Igfbp4,Iqgap2,Ksr1,lnc1716,lnc21125,lnc29699,
lnc33081,lnc35020,lnc5921,lnc7860,lnc8555,lnc8568,lnc8627,lnc8639,lnc8658,lnc8863,lnc8970,Loxl3,Lrrc8c,
Macrod1,Mamld1,Myo1e,Ncoa7,Nsd3,Osbpl1a,Pam,Parp8,Plscr4,Prkag2,Prkch,Rarg,Rnf165,Trerf1,Trip4,Zeb2,Zfpm2

1300017J02Rik,Acsm5,Adtrp,Amy1,Aqp9,Bbox1,Car5a,Cfi,Chuk,Cpn1,Cry2,Dap,Echdc3,
F12,Hgfac,Hlf,Ikbkg,Larp1b,lnc10151,lnc1018,lnc11590,lnc12046,lnc14604,lnc14605,lnc15262,
lnc1550,lnc2342,lnc33230,lnc41044,lnc41384,lnc43327,lnc4334,lnc4351,lnc4362,lnc43856,
lnc47387,lnc47625,lnc5911,lnc6546,lnc7290,lnc7375,lnc7523,lnc7649,lnc8053,lnc8940,
Map3k13,Otc,Pemt,Pex19,Rcl1,Sec14l4,Sec16b,Selenbp2,Sgk2,Them7,Wdtc1,Aasdh,
Atoh8,C130074G19Rik,Cth,Cyp2a12,Dclk3,Emc1,Eppk1,Fabp12,Fbxw9,Foxa2,Ggcx,
Gm47904,Hnf1a,Htatip2,Ice2,Insc,lnc1057,lnc12365,lnc12630,lnc12633,lnc12771,
lnc13511,lnc13515,lnc14302,lnc14755,lnc1602,lnc17255,lnc17289,lnc18831,lnc19399,
lnc20789,lnc21288,lnc22126,lnc26884,lnc31725,lnc31727,lnc36873,lnc40479,lnc40940,
lnc43540,lnc4370,lnc5261,lnc5356,lnc6061,lnc6501,lnc9709,lnc981,lnc987,Lrp4,Mst1,
Ovol1,Pkp2,Slc10a5,Smad9,Sox5,Sox7,Suds3,Tead3,Trpv1,Ugt1a1,Wnt10a,Zfp612

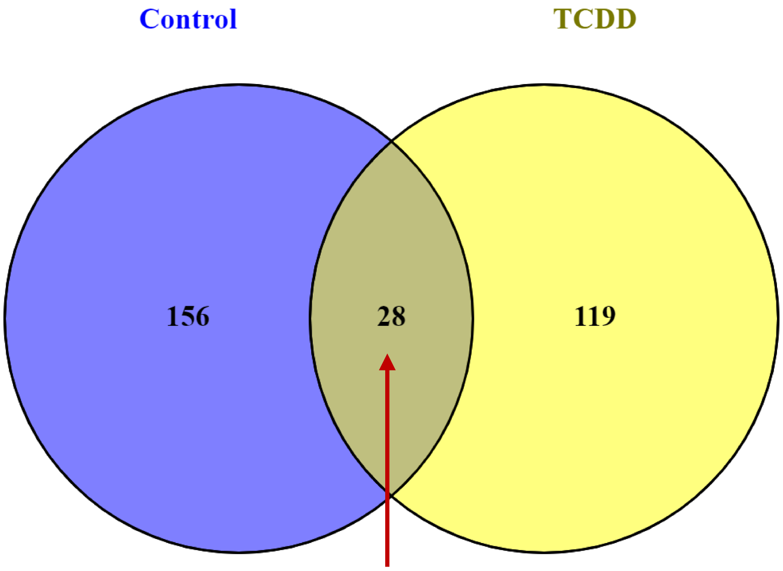

Acsm1,Akap1,Cat,Creb3l3,Echdc2,Erbb3,Gm10768,Hnf4a,Hpn,lnc12772,
lnc1966,lnc23914,lnc6166,lnc8349,lnc979,Mttp,Nr1i2,Pcx,Pex14,Ppara,
Sdc4,Sirt3,Slc25a42,Slc38a3,Tfr2,Timd2,Tmprss6,Upb1

Fig. S6
Control
network

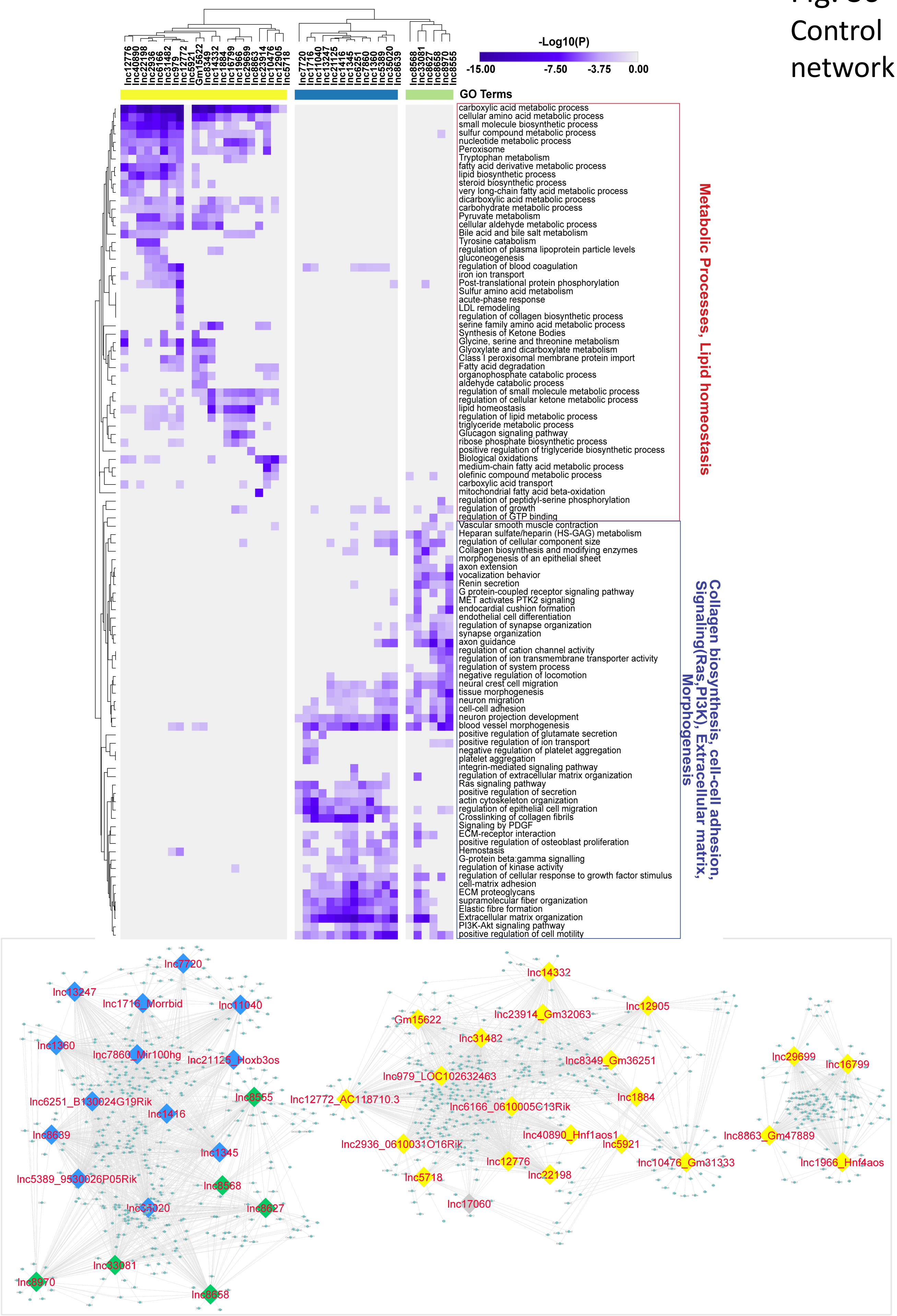

Fig. S7
TCDD
network

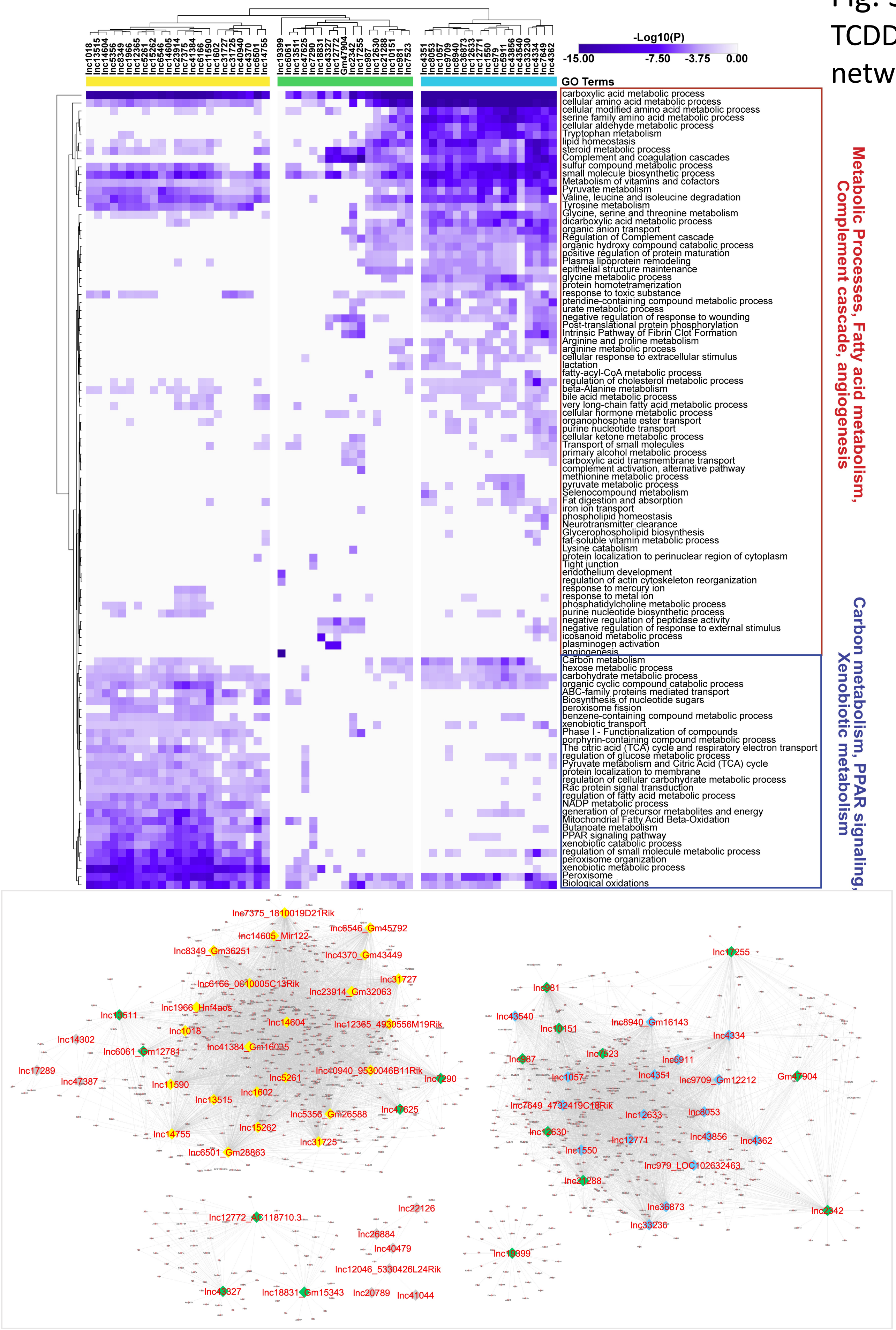

Fig. S8
Control +
TCDD

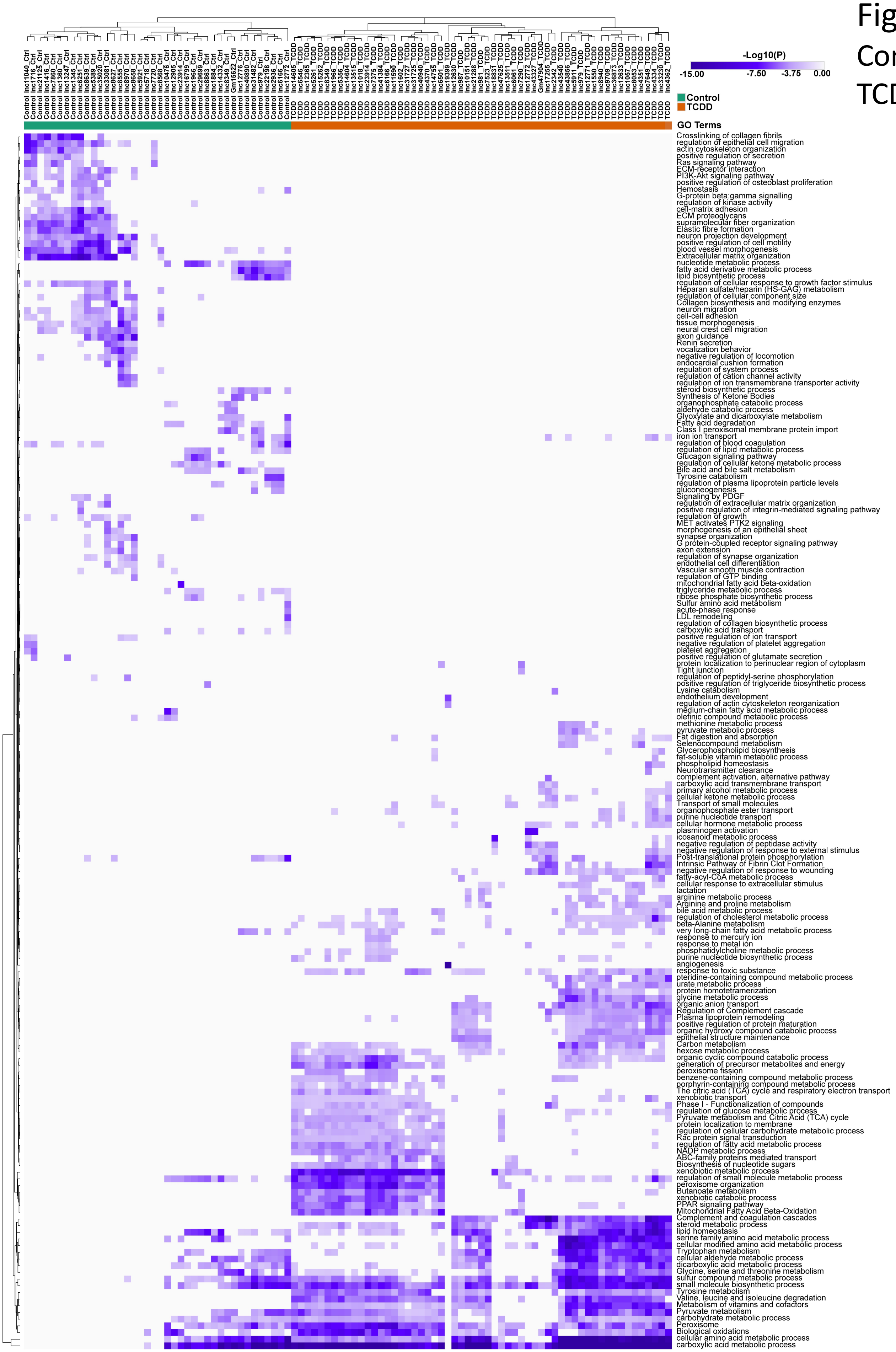

Supplement: Supplement 1 — Fig. S1. A. Mouse liver cell subpopulations and cell-type specific markers. A. UMAP displaying clusters of liver nuclei clusters based on 15,573 single-nuclei transcriptomes integrated from all four snRNA-seq samples, two control liver and two TCDD liver snRNA-seq samples (biological replicates). Cell count values for each cell type and for each condition (C: control; T: TCDD) are indicated in square brackets. B. Dot plot showing average expression values for marker genes (shown on X-axis) across the eight hepatic cell clusters (identified on y-axis) for liver nuclei aggregated from all 4 samples -- control and TCDD. Fig. S2. Gene induction and gene repression responses to TCDD. Shown are bar plots indicating the total number of up regulated (green) and down regulated genes (red). (A), all genes, (B) protein-coding genes, and (C) lncRNA genes, in each of the 8 indicated liver cell types, at |fold-change| >4 and FDR <0.05. Many fewer genes were up regulated than were down regulated in several of the NPC clusters. (D) Pie charts showing numbers of lncRNAs and protein-coding genes that are differentially expressed in TCDD-exposed vs control liver in hepatocytes only, in non-parenchymal cells (NPCs) only, or is both hepatocytes and NPCs based on data shown in Table S1, columns AO and AP. Fig S3. Violin plots and feature plots showing expression data for select TCDD-responsive genes. A. Violin plots for Cyp1a1, a classic TCDD/Ahr response gene that is strongly induced by TCDD in multiple liver cell types, and for two lncRNAs whose expression is highly inducible in cholangiocytes but not in other liver cell types. B. Feature plots showing expression data for two lncRNAs that are highly induced by TCDD in hepatocytes but not in other cell clusters. Expression data is presented superimposed on the UMAP of cell clusters shown in Fig. S1A, where the individual cell clusters are identified. C. Expression data for lncRNAs repressed by TCDD. Top panel of C, violin plots showing [file NIHPP2023.01.07.523119v1-supplement-1.pdf]
